# Unusual markedly-dilated chorionic vessels with placentomegaly

**DOI:** 10.1186/2193-1801-3-146

**Published:** 2014-03-17

**Authors:** Yoshitaka Kaido, Akihiko Kikuchi, Tomonobu Kanasugi, Rie Oyama, Toru Sugiyama

**Affiliations:** Department of Obstetrics and Gynecology, Iwate Medical University School of Medicine, 19-1 Uchimaru, Morioka, Iwate, 020-8505 Japan

**Keywords:** Dilation of chorionic vessels, Placentomegaly, Fetal anemia, Fetal growth restriction

## Abstract

We reported a very rare case with placentomegaly and markedly-dilated chorionic vessels. A 23-year-old Japanese pregnant woman was referred to our hospital at 32 weeks of gestation because of suspected dilatation of the umbilical vein. Ultrasound revealed fetal growth restriction with no fetal anomalies. The placenta was thick. The umbilical cord had two arteries and one vein, and marginal insertion of the umbilical cord in the placenta was suspected. A lot of remarkably tortuous tubular structures were observed on the surface of the placenta. Slow blood flow, indemonstrable with color Doppler, was observed within them. Labor started suddenly and progressed very rapidly at 33 weeks of gestation. A female infant weighing 1524 g was delivered. The infant had no malformations. However, she showed hypotension, anemia and DIC and required blood transfusion. The placenta was very large, weighing 1416 g. On the fetal surface, numerous dilated and tortuous vessels were observed, arising from a vein that was connected to the umbilical vein. These venous channels were dilated and tortuous and led into the placenta. In conclusion, prenatal diagnosis of placentomegaly and markedly-dilated chorionic vessels requires assessment of growth, anemia and DIC of the fetus and the newborn infant.

## Introduction

Placentomegaly and dilation of chorionic vessels are rare anomalies of the placenta. There have been limited papers that reported on anomalies of the chorionic vessels, although venous anomaly of those is clinically important because it may threaten the life of the fetus. Recently, placental mesenchymal dysplasia (PMD) has been reported as a benign entity of the placenta (Taga et al. 2013). PMD was described for the first time by Moscoso et al. in 1991 (Moscoso et al. 1991). It is characterized by placentomegaly with hydropic villi and is often accompanied by a dilation of chorionic vessels (Moscoso et al. 1991; Sander 1993; Kinoshita et al. 2007; Koga et al. 2013). As clinical symptoms of the fetus, fetal growth restriction (FGR) (Sander 1993; Kinoshita et al. 2007; Koga et al. 2013) and hematologic disorders (Sander 1993; Koga et al. 2013) have been reported in PMD. PMD is often suspected by prenatal ultrasound showing cystic lesions in the placenta similar to those of partial mole.

We report a very rare case with placentomegaly and markedly-dilated chorionic vessels, without cystic lesions in the placenta typical of PMD. As far as we know, only one case exactly like ours has been reported so far (Lee et al. 1991).

## Case report

A 23-year-old gravida 0, para 0, Japanese pregnant woman was referred to our hospital from another hospital at 32 weeks of gestation because of suspected dilatation of the umbilical vein. The patient had no past or familial medical history, and her pregnancy had been uneventful except for pregnancy-induced hypertension (PIH). Her blood pressure was 149/92 mmHg; urinary protein, 2+; and her lower legs were edematous.

**Figure 1:**
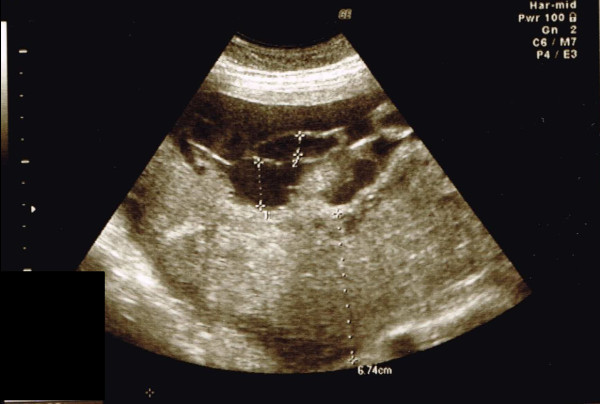
**Ultrasound at 32 weeks’ gestation showing a thick placenta and tortuous tubular structures on the surface of the placenta.** No cystic lesions were observed in the parenchyma of the placenta.

**Figure 2:**
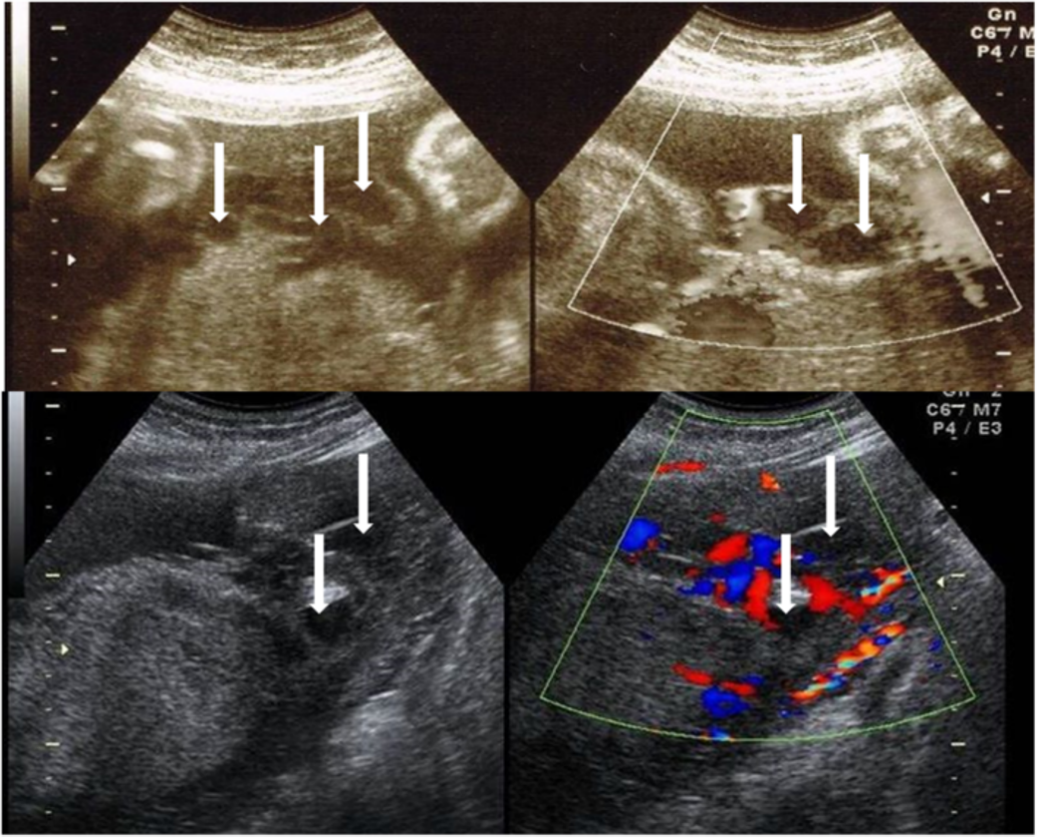
**Ultrasound at 32 weeks’ gestation showing a lot of remarkably tortuous tubular structures (arrows on the left pictures) on the surface of the placenta.** Slow blood flow, indemonstrable with color Doppler, was observed within them (arrows on the right pictures).

**Figure 3:**
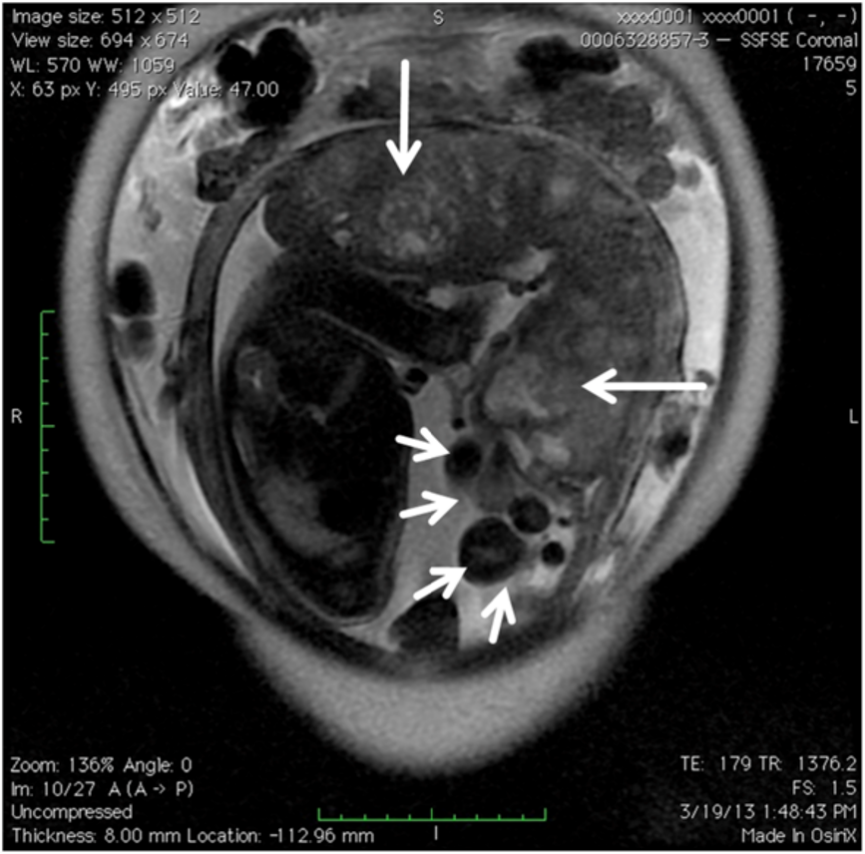
**MRI at 32 weeks’ gestation clearly indicated numerous dilated and tortuous vessels (short arrows) on the surface of the placenta.** The placenta was thickened (long arrows) with no cystic lesions in the parenchyma.

Obstetric abdominal ultrasound revealed a fetus in cephalic presentation with an estimated fetal weight of 1498 g (−1.7 standard deviation), an amniotic fluid index of 10.6 cm, resistance index of the umbilical artery of 0.65, resistance index of the middle cerebral artery of 0.87, peak systolic velocity of the middle cerebral artery of 60.2 cm/s (<1.5 MoM). No fetal anomalies were found. The placenta was thick and located on the posterior uterine wall. The umbilical cord had two arteries and one vein, and marginal insertion of the umbilical cord in the placenta was suspected. A lot of remarkably tortuous tubular structures were observed on the surface of the placenta (Figure 1). Slow blood flow, indemonstrable with color Doppler, was observed within them (Figure 2). Magnetic resonance imaging (MRI) clearly indicated numerous dilated and tortuous vessels on the surface of the placenta (Figure 3). Transvaginal ultrasound revealed normal cervical length. Cardiotocogram showed a reassuring fetal status.

The patient was admitted to our hospital for management of PIH. Although her condition of PIH was stable, labor started suddenly and progressed very rapidly at 33 weeks of gestation. A female infant weighing 1524 g was delivered vaginally with an Apgar scores of 8 and 9 at 1 and 5 min, respectively.

The infant had no malformations. She was admitted to the NICU for a preterm infant without incubation and oxygen supply. However, her blood pressure was 39/21 mmHg; hemoglobin content, 10.1 mg/dl; platelet count, 71000/μl; fibrinogen, 49.2 mg/dl; FDP, 21.0 μg/ml; and D-D, 8.8 μg/ml. Because these data met the criteria of neonatal DIC, she required blood transfusion and administration of dopamine hydrochloride. Her general condition gradually recovered and she was transferred to a nearby hospital from her parents’ home on the 32nd day after birth.

**Figure 4:**
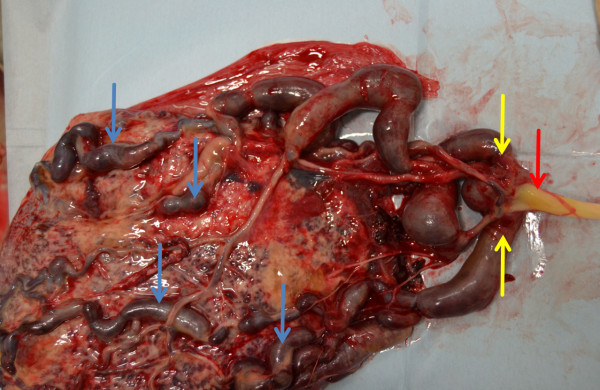
**Delivered placenta.** The umbilical cord was inserted in a velamentous fashion (red arrow). The fetal surface of the placenta showed numerous dilated and tortuous vessels (blue arrows) on and under the chorionic membranes. These vessels branched from the umbilical vein (yellow arrows).

**Figure 5:**
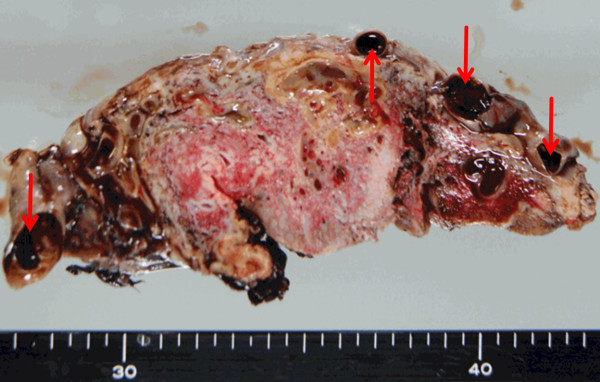
**Cut surface of the delivered placenta.** Thrombi (red allows) were observed in the dilated vessels on the fetal surface of the placenta. There were no cystic lesions in the parenchyma of the placenta.

The placenta was very large, weighing 1416 g. On the fetal surface, numerous dilated and tortuous vessels were observed, arising from a vein that was connected to the umbilical vein (Figure 4). These venous channels were dilated and tortuous and led into the placenta. Velamentous insertion of the umbilical cord was present and Wharton’s jelly was deficient near the insertion site. Thrombi were observed in the dilated vessels on the fetal surface of the placenta (Figure 5).

**Figure 6:**
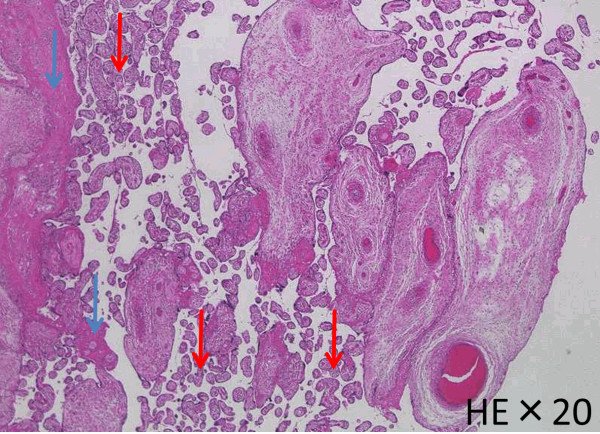
**Parenchyma of the placenta.** The villi were normally formed (red allows) and hydropic villi were not observed. Part of the intervillous spaces were occluded by precipitated fibrin (blue allows), compatible with PIH.

**Figure 7:**
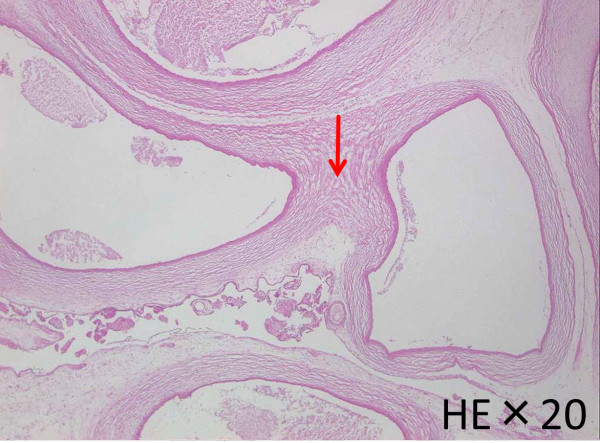
**Intraplacental vessels were extremely dilated and walls of these vessels were fused (red arrow).**

Histologic examination revealed necrosis in the decidua. The villi were normally formed and hydropic villi were not observed (Figure 6). Part of the intervillous spaces were occluded by precipitated fibrin and the villous stroma were edematous. Infarction was widely noted in the amnion. Intraplacental vessels were extremely dilated and walls of these vessels were fused (Figure 7). On the other hand, the umbilical arteries and vein, and parenchyma of the maternal side of the placenta had no anomalies.

## Discussion

We have found the only one previous report of a case with placentomegaly and dilation of the chorionic vessels of the placenta exactly like ours (Lee et al. 1991). Although some of the papers on PMD regarded it as a case of PMD (Kinoshita et al. 2007; Koga et al. 2013), Lee et al. described that the villi in their case were normally formed microscopically. Similarities in pathologic and clinical manifestations between the case reported by Lee et al. and our case include; (1) numerous dilated and tortuous vessels on fetal the surface of the placenta, (2) very large and heavy placenta, (3) fetal growth restriction, and (4) anemia in the infant.

The chorionic vessels are supposedly formed by the branching of the umbilical vessels (Lee et al. 1991). As the direction of flow from the fetus is established, a minimum number of necessary vessels remain, and the balance of the vascular plexus disappears (Lee et al. 1991). This regressive process may be halted or interrupted by indefinite factors. So it is thought that placental vascular abnormality occurs very early in embryonic life (Sander 1993; Kinoshita et al. 2007; Lee et al. 1991). However, Jauniaux et al. described that the dilation of chorionic vessels is not observed until the second trimester and becomes characteristic during the third trimester (Jauniaux et al. 1997). In our case, therefore, dilation of chorionic vessels may be considered to have progressed gradually secondary to peripheral villous vascular obstruction. Circulatory failure due to these abnormal blood vessels and thrombosis may have caused enlargement of the placenta compensatively. But on the other hand, this explanation might not be enough because veins usually dilate not at the central site but at the peripheral site of their obstruction.

Sander described that the cause of FGR might be the diversion of blood away from the maternal intervillous space and that fetal anemia might be a result of a microangiopathic process occurring within the aberrant placental vasculature (Sander 1993). In addition, thrombosis in chorionic vessels might have caused FGR associated with fetoplacental circulatory insufficiency in our case.

In conclusion, we reported a very rare case with placentomegaly and markedly-dilated chorionic vessels. Prenatal diagnosis of this condition using ultrasound and MRI requires assessment of growth, anemia and DIC of the fetus and the newborn infant.

## Consent

Written informed consent was obtained from the patient for the publication of this report and any accompanying images.
